# Adding MRI as a Surveillance Test for Hepatocellular Carcinoma in Patients with Liver Cirrhosis Can Improve Prognosis

**DOI:** 10.3390/biomedicines11020382

**Published:** 2023-01-27

**Authors:** Su Jong Yu, Jeong-Ju Yoo, Dong Ho Lee, Su Jin Kim, Eun Ju Cho, Se Hyung Kim, Jeong-Hoon Lee, Yoon Jun Kim, Jeong Min Lee, Jae Young Lee, Jung-Hwan Yoon

**Affiliations:** 1 Department of Internal Medicine and Liver Research Institute, College of Medicine, Seoul National University, Seoul 03080, Republic of Korea; 2 Department of Internal Medicine, Soonchunhyaung University, Bucheon Hospital, Bucheon 14584, Republic of Korea; 3 Department of Radiology and Institute of Radiation Medicine, College of Medicine, Seoul National University, Seoul 03080, Republic of Korea; 4 Department of Statistics, Soonchunhyaung University, Bucheon Hospital, Bucheon 14584, Republic of Korea

**Keywords:** MRI, surveillance, hepatocellular carcinoma, prognosis

## Abstract

Gadoxetic acid disodium (Gd-EOB-DTPA)-enhanced magnetic resonance imaging (MRI) can detect early stages of hepatocellular carcinoma (HCC). However, the survival benefit of Gd-EOB-DTPA-enhanced MRI in the surveillance of patients with cirrhosis has not yet been determined. We explored whether the intermittent replacement of ultrasonography (USG) with Gd-EOB-DTPA-enhanced MRI during HCC surveillance improved the clinical outcomes of patients with cirrhosis. We performed a retrospective cohort study of 421 HCC patients who were newly diagnosed during surveillance. Of these patients, 126 (29.9%) underwent surveillance based on Gd-EOB-DTPA-enhanced MRI and USG (USG+MRI group). The patients (295, 70.1%) who did not undergo MRI during surveillance were referred to as the USG group. In the USG+MRI group, 120 (95.2%) of 126 patients were diagnosed with early-stage HCC, whereas 247 (83.7%) of 295 patients were diagnosed with early-stage HCC in the USG group (*P* = 0.009). The significantly longer overall survival and time to progression in patients in the USG+MRI group compared to the unmatched cohort USG group was consistently observed by inverse probability weighting and propensity score-matched analysis. Gd-EOB-DTPA-enhanced MRI combined surveillance improved the detection of early-stage HCC and clinical outcomes such as overall survival and the time to progression in patients with cirrhosis.

## 1. Introduction

Most guidelines insist surveillance for hepatocellular carcinoma (HCC) of high-risk patients with chronic liver diseases by ultrasonography (USG) every 6 months [with or without alpha-fetoprotein (AFP)] [1,2]. However, the ideal imaging modality for the detection of HCC is still controversial. Although USG is the most widely used surveillance tool due to its reasonable price and good accessibility, [1,2] there is a risk of missing lesions due to low sensitivity to small nodules. Accuracy of USG examination is reported in various ways depending on the equipment, transducer or operator.

In practice, some providers recommend using computed tomography (CT) for HCC detection, which is more sensitive but less specific compared to USG [3]. However, given the higher cost, increased false-positive result rate, and radiation exposure, CT is not currently recommended as a regular surveillance option for HCC in high risk patients [4].

Magnetic resonance imaging (MRI) has been well shown as an alternative tool to multi-detector CT (MDCT) examination because of the enhanced imaging capabilities and free of radiation [5]. Gadoxetic acid disodium (Gd-EOB-DTPA) is specified for liver MR imaging with hepatocyte-specific properties, allowing for the acquisition of hepatobiliary phase images [6,7,8,9]. In addition, Kim et al. published that Gd-EOB-DTPA-enhanced MRI for surveillance resulted in an increased HCC detection and decreased false-positive findings compared to USG at high risk patients for HCC [10]. Moreover, surveillance every 6 month using liver-specific contrast MRI might be cost-effective compared with USG [11]. However, whether surveillance by Gd-EOB-DTPA-enhanced MRI will improve the clinical outcome compared to that of conventional surveillance by USG is unknown.

The aim of this study was to evaluate whether Gd-EOB-DTPA-enhanced MRI for surveillance improved clinical outcomes by the sensitive detection of early-stage HCC compared to standard surveillance based on USG in cirrhotic patients.

## 2. Materials

### 2.1. Patients

Patients was derived from 3422 consecutive cirrhosis patients who were followed for HCC surveillance at Seoul National University Hospital, an academic tertiary hospital in Seoul, Korea, between January 2008 and August 2013. Of these, 444 patients were diagnosed with HCC during surveillance at our institution. Patients were excluded if they had one of the following reasons: follow-up loss before enrollment (*n* = 2), no HCC at enrollment (*n* = 1), a short follow-up period less than 6 months (*n* = 3), or who performed additional Gd-EOB-DTPA-enhanced MRI for patients with CT-confirmed HCC (*n* = 17). The diagnosis of cirrhosis was histologically proved or made on according to commonly accepted standards imaging studie [12]. This study was approved at the Institutional Review Board of Seoul National University Hospital (IRB number 1312-064-541), and conformed to the ethical guidelines of the World Medical Association Declaration of Helsinki. Informed consent was waived from the IRB due to the retrospective design.

### 2.2. Image Evaluation/Surveillance Strategy

**Figure 1:**
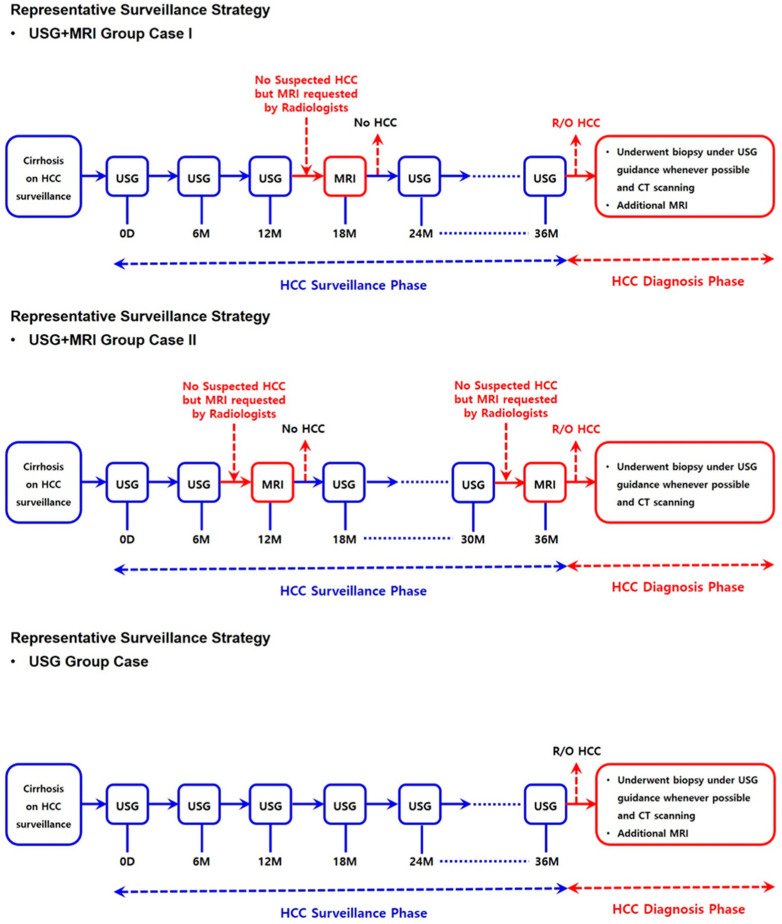
Representative surveillance strategy of each group. (USG, ultrasound; HCC, hepatocellular carcinoma; MRI, magnetic resonance imaging; CT, computed tomography).

Representative surveillance strategy of each group is shown in Figure 1. In our institution, clinicians generally conduct USG for initial surveillance imaging with alpha fetoprotein (AFP), and all patients were educated on the significance of attending regular follow-up UGS and AFP at least every six months [13]. Gd-EOB-DTPA-enhanced MRI was first used in our institution from January 2008 and was gradually incorporated for evaluation of HCC.

In this study, MRI was used for surveillance, not for diagnostic purposes. MRI was not performed according to a predetermined protocol (e.g., once every 1–2 years). Instead, as described below, MRI scans were recommended only when USG showed suboptimal quality. Thus, MRI was performed 1–2 times on average.

In all patients including MRI group, there was no focal lesion suspected of HCC or dysplastic on previous USG. Instead, MRI was performed if the previous ultrasound image was suboptimal, such as LI-RADs surveillance quality grade C. In this suboptimal quality of USG surveillance, abdominal radiologists recommend MRI for next imaging surveillance tool. This is because the sensitivity of the USG to detect HCC is suboptimal, and the risk of HCC was high in our patients group (hepatocellular carcinoma risk index 2.7; cumulative incidence of HCC at 4 years, more than 30.1%) [11,14]. Thus, the decision to conduct MRI as the next imaging modality during surveillance was not based on patient characteristics but was a matter of protocol adoption. During the study period, there was no change in the method or modality of HCC management at our institution.

### 2.3. Acquisition of MRI Images

MR images were obtained by either a 1.5T (Signa HDx, GE Medical Systems, Milwaukee, WI, USA) or a 3.0T (Signa Excite, GE Medical Systems; Verio, Siemens Medical Solutions, Erlangen, Germany; Trio, Siemens Medical Solutions) superconducting system using either an 8-channel (Signa HDx, Excite) or a 32-channel (Verio, and Trio) phased-array coil. All patients received a rapid bolus of gadoxetic acid (Primovist^®^; Bayer Healthcare, Berlin, Germany) followed by a 30 mL saline flush. The scanning delay times for arterial phase imaging were determined with real-time MR fluoroscopic monitoring after contrast administration. The arterial phase was scanned 7 s after the contrast media arrived at the thoracic aorta, and the portal venous phase, late dynamic phase, and hepatobiliary phase were subsequently scanned 50 s, 3 min, and 20 min after, respectively. The acquisition of USG and CT is described in the Supplementary Material.

### 2.4. Image Analysis and Diagnostic Criteria

All images were interpreted as a part of routine clinical practice by nine board-certified abdominal radiologists with substantial expertise in liver imaging using a picture archiving and communication system.

The confirmation of HCC was based on the results of the histologic examination and/or typical findings from dynamic 4-phase CT images and/or liver MRI recommended by practice guidelines [15,16,17,18,19]. We used the EASL [17], AASLD [16], and KLCA [18] guidelines for the diagnosis of HCC. In Gd-EOB-DTPA-enhanced MRI, dynamic scanning stage recommended in each guideline was used. In the EASL or AASLD criteria, only the arterial phase and portal venous phase are used for the diagnosis of HCC, and late dynamic phase or hepatobiliary phase washout are not recognized as diagnostic criteria. On the other hand, in the KLCA-HCC guideline, all of the above-mentioned dynamic scanning, T2 weighted image, and diffusion weighted image are used for imaging diagnosis of HCC. All patients were staged according to the Barcelona Clinic Liver Cancer staging system (BCLC) staging system [20]. The risk of HCC at the time of HCC diagnosis was calculated by the index formula as follows: risk Index = 1.65 (if the prothrombin activity is ≤75%) + 1.41 (if the age is 55 years or older) + 0.92 (if the platelet count is ≤75 × 10^3^/mm^3^) + 0.74 (if the anti-HCV or HBsAg test is positive), and high risk was defined as a risk index greater than 2.33 [21].

### 2.5. Outcomes and Follow-Up Assessment

The primary outcome was overall survival (OS) defined as the time from the first surveillance test to any cause of death. The secondary outcome was the time to progression defined as from the first surveillance test to the first cancer progression.

We followed up with patients via the routine protocol. Biochemical test and CT imaging was performed every 3 months after initial treatment. Two years after HCC was cured, the examination interval was extended to 3–6 months. For the evaluation of treatment response, modified Response Evaluation Criteria in Solid Tumors (mRECIST) was used [22].

### 2.6. Statistical Analysis

Continuous variables were compared using the Student’s *t*-test and categorical variables were analyzed by Chi-squared test. Survival analysis and comparison was calculated by the log-rank test or Kaplan-Meier method. Factors that influenced survival was analyzed by Cox proportional hazards model.

To minimize lead-time bias [23], the lead time was calculated in the USG+MRI group using Schwartz’s formula [24], originally proposed for calculating tumor growth

*t* = DT × 3 × log(*d*_1_/*d*_0_)/log(2) (1)

where *t* is the lead time (days), DT is the median value of the tumor volume doubling-time (days) proposed by Scheu et al. [25], *d*_0_ is the median tumor diameter in the USG+MRI group, and *d*_1_ is the median tumor diameter in the USG group. The calculated lead time for the USG+MRI group was subtracted from their survival values.

In order to minimize the difference in the underlying characteristics of the two groups, we used inverse probability treatment weighting (IPTW) with propensity scores [26]. To derive the propensity scores, the same three adjustment variables (age, gender, and liver function) as for the multivariable Cox proportional hazards model were used. Propensity score matching method was additionally performed for sensitivity analysis, The details of the analytic method are described in the Supporting information.

All statistical analyses were performed using R software (version 3.0; http://cran.r-project.org/). *P* < 0.05 was considered significant.

## 3. Results

### 3.1. Characteristics of the Study Population

This study included 421 patients in total. The USG group consisted of 295 (70.1%) patients with HCC detected during surveillance based on ultrasonography, and the USG+MRI group consisted of 126 (29.9%) patients with HCC detected during surveillance based on Gd-EOB-DTPA-enhanced MRI and ultrasonography. In the USG + MRI group, the median number of MRI surveillance was 1 (min 1, max 4). The median interval of surveillance between two groups showed no significant difference (6.5 vs. 5.4 months; *P* = 0.461).

The patients in the USG and USG+MRI groups shared comparable baseline charactericstics (Table 1), including age (>60 years 65.8% vs. 56.3%), sex (male 71.5% vs. 75.4%), and liver function patients (Child-Turcotte-Pugh class A 78.0% vs. 82.5%). The number of chronic hepatitis patients were 209 patients in the USG group and the 99 patients in the USG+MRI group. Of these, 141 (55.1%) and 127 (51.4%) were taking an antiviral agent (*P* = 0.41). In terms of HCC risk, the mean risk index value was similar between the USG and USG+MRI groups (2.8 ± 1.1 vs. 2.6 ± 1.1, respectively; *P* = 0.217).

### 3.2. Stage of HCC and Treatment Method

Early-stage HCC (defined as BCLC stages 0 & A) was significantly higher in the USG+MRI group than in the USG group (*n* = 120, 95.2% vs. *n* = 247, 83.7%; *P* = 0.009) (Table 1). In the evaluation of cancer stages other than BCLC stages, rate of early HCC was significantly greater in the USG + MRI group (Supplementary Table S1). In the UG+MRI group, more HCCs were within the Milan criteria compared to the USG group (*n* =117, 92.9% vs. *n* = 250, 84.7%, respectively; *P* = 0.009). The proportion of patients who achieved complete response after first treatment was greater in the USG + MRI group than the USG group (*n* = 92, 31.2% vs. *n* = 55, 43.7%, respectively; *P* = 0.019; Table 1). Initial treatment modality between the groups is presented in Supplementary Table S1.

### 3.3. Prognosis of the Patients

The median follow-up period was 92 months (interquartile range [IQR], 43–135 months) and 78 months (IQR, 44–144 years) for the USG and USG+MRI groups, respectively (*P* = 0.727; Table 1). During the observational period, 110 (26.1%) patients died.

**Figure 2:**
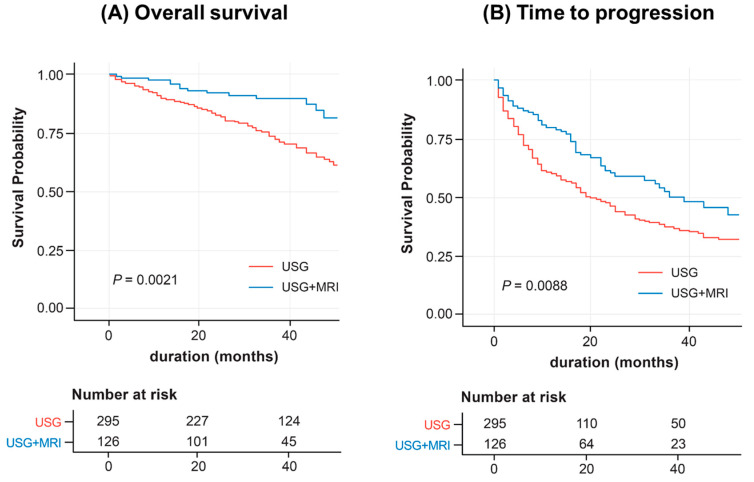
Overall survival and time to progression in patients surveilled by USG alone or USG and Gd-EOB-DTPA-enhanced MRI (unmatched cohort). (**A**) Overall survival. (**B**) Time to progression.

Overall survival rates was signicantly higher in the USG+MRI group than the USG group in Kaplan-Meier analysis (*P* = 0.002; Figure 2A). The time to progression was significantly longer in the USG+MRI group than in the USG group (*P* = 0.008; Figure 2B).

The USG+MRI group had a significant lower risk of overall mortality (hazard ratio [HR] = 0.56; 95% confidence interval [CI]: 0.32–0.99; *P* = 0.047) according to multivariate Cox proportional hazards model (Table 2). The USG+MRI group showed a longer time to progression (HR = 0.76; 95% CI: 0.55–1.04; *P* = 0.091) than the USG group, but it was not significant (Table 3). To avoid lead-time bias, the lead time using a formula proposed by Schwartz was calculated [24]. When two different times, 117 days and 60 days, were assumed as median tumor volume-doubling times, the calculated lead times were 3.8 months and 1.9 months, respectively, and the 5-year survival of the USG+MRI group remained longer than that of the USG group (Supplementary Figure S1; *P* = 0.011, respectively).

### 3.4. Inverse Probability Weighting and Sensitivity Analysis

**Figure 3:**
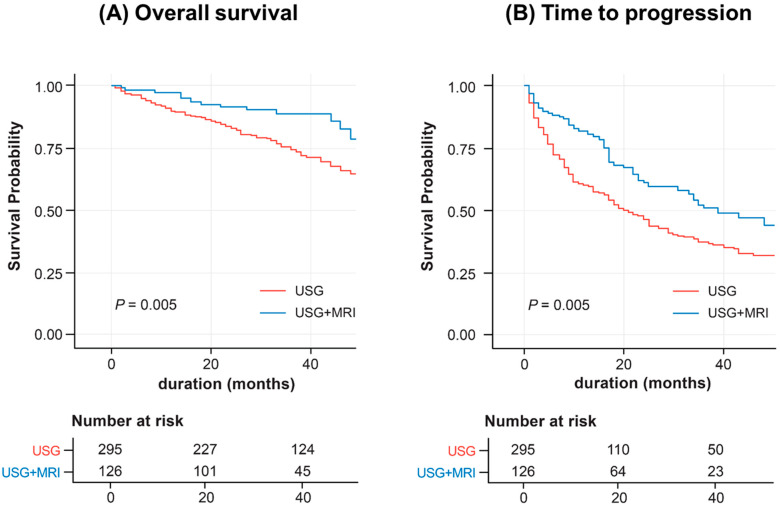
Overall survival and time to progression of patients surveilled with USG alone or USG and Gd-EOB-DTPA-enhanced MRI (IPTW). (**A**) Overall survival. (**B**) Time to progression.

Next, we performed an inverse probability weighting (IPTW) analysis to adjust for confounding factors and baseline characteristics. After IPTW, the baseline characteristics of the two groups were well balanced (Supplementary Figure S2). In the Kaplan-Meier curve using IPTW, the overall mortality of the USG+MRI group was significantly lower, and time to progression was longer (log-rank *P* = 0.005) than the USG group (Figure 3). Furthermore, time to progression was significantly longer in the USG+MRI group in multivariate analysis (Supplementary Table S2; HR = 0.70; 95% CI: 0.51 –0.95; *P* = 0.024). However, the overall survival was not significantly different in the USG+MRI group in IPTW analysis (Supplementary Table S3; HR = 0.72; 95% CI: 0.43 –1.19; *P* = 0.209).

Sensitivity analysis was performed by utilizing propensity score matching (PSM), and had similar results with IPTW analysis. Baseline characteristics of PSM cohort (Supplementary Table S4) and the Kaplan-Meier curve of overall survival (Supplementary Figure S3A) and time to progression (Supplementary Figure S3B) is presented in Supplementary material.

## 4. Discussion

This study aimed to evaluate whether performing Gd-EOB-DTPA-enhanced MRI with a liver-specific contrast media during HCC surveillance enhanced the clinical outcomes in patients who had previously undergone ultrasonography-based surveillance programs. According to our study, an additional scan of Gd-EOB-DTPA-enhanced MRI might increase the sensitivity of early-stage HCC, which led to improved post-treatment outcomes. Even after correcting for lead-time bias, the time to progression and overall survival of the patients were significantly longer in the USG+MRI group than in the USG group, with the results being consistent in the unmatched cohort, inverse probability weighting and propensity score-matched analyses. We believe that the USG + MRI group had high HCC detection rates in the early stage, which led to a high CR achieving rate after the initial treatment, and resulted in an improvement on overall survival.

Recent study supports our hypothesis that HCC surveillance using Gd-EOB-DTPA-enhanced MRI is superior to USG to detect early HCC [10]. According to the previous study, MRI surveillance yielded a detection rate of 84.8%, significantly higher than the rate of 27.3% by USG, for detecting very early-stage HCC (single lesion size of <2 cm) [10]. However, the previous study included no control group that underwent ultrasonography surveillance only. In addition, the previous study could not show whether HCC surveillance under Gd-EOB-DTPA-enhanced MRI would reduce mortality.

The higher accuracy of Gd-EOB-DTPA-enhanced MRI was also proven by a meta-analysis [27] and was interpreted as the superior accuracy for MRIs detecting small HCC lesions by the hepatobiliary phase [28,29]. Our results are also relevant since at least a third of early HCC recurrences may represent pre-existing dissemination of the primary tumor that was not found at initial treatment [30,31,32]. Even among patients with a single-nodular HCC on dynamic CT images, a supplementary examination via Gd-EOB-DTPA-enhanced MRI increased the detection of additional HCC nodules by 16% in the study population [33]. The addition of Gd-EOB-DTPA-enhanced MRI in the hepatobiliary phase statistically increased the accuracy of HCC diagnosis [34]. In addition to the sensitive diagnostic findings of HCC by Gd-EOB-DTPA-enhanced MRI, the detection of non-hypervascular nodules in the hepatobiliary phase may suggest increased hepatocarcinogenesis [35]. A recent study reported that non-hypervascular nodules large than 1 cm (41.2%, 14/34) progressed into overt HCC at a significantly higher rate than nodules smaller than 1 cm [36]. A significant proportion of non-hypervascular hepatobiliary phase nodules bigger than 1cm showed pathologically malignant features or newly developed hypervascularity during the follow-up period [37].

Since the hepatic features of liver cirrhosis such as fibrous septa and regenerative nodules make ultrasonographic evaluation difficult, an ultrasonography-based surveillance program for early-stage HCC is insufficiently sensitive for many cirrhosis patients [3,38] making our findings clinically meaningful. A recent meta-analysis noted that the pooled sensitivity and specificity of ultrasonography in detecting HCC of any stage was 94%. However, the sensitivity decreased to 63% in detecting the early stages of HCC [39]. In cirrhosis patients, the sensitivity of ultrasonography alone in HCC detection was even lower (32%) [40]. Indeed, the lack of HCC detection by ultrasonography accounted for 70% of the surveillance failures in the Hepatitis C Antiviral Long-term Treatment Against Cirrhosis trial [41].

The sensitivity of ultrasonography is suboptimal, especially in the nodular liver with multiple dysplastic nodules [42]. The sensitivity of ultrasonography for HCC detection is also significantly associated with patient characteristics; it is more difficult in obese patients with poor hepatic windows. Although ultrasonography resolution has been much improved recently, the experience of the examiner influences the accuracy of liver structure evaluation. Examination of a cirrhotic liver for the detection of HCC must be done by highly experienced and qualified radiologists due to its variable appearances on ultrasonography.

As mentioned above, MRI is obviously advantageous for early tumor detection. However, no studies have shown that such early tumor detection leads to an increase in overall survival. The most important strength of this study is that it was the first study to show that the use of MRI in surveillance tests increased overall patient survival. In particular, it is clinically meaningful that the superiority of USG+MRI was demonstrated in corrected overall survival analysis or IPTW analysis. Notably, the superiority of USG+MRI disappeared in the presence of tumor stages in the multivariate analysis, indicating that surveillance imaging modality interacts with the prognosis via its effect on the stages of HCC. In fact, further analysis revealed that there was an interaction between the tumor stage and surveillance imaging modality (Supplementary Table S5).

The major limitation of this study was that it was based on observational data. Our results should be confirmed by a prospective study comparing Gd-EOB-DTPA-enhanced MRI and ultrasonography for HCC surveillance. Moreover, the results should be interpreted with some reservation because of the potential selection bias in the direction of more meticulously followed-up cases in the USG+MRI group. However, the mean HCC risk index value did not show any significant difference between the ultrasonography group and the USG+MRI group. Furthermore, the goal of this study is not to support the view that Gd-EOB-DTPA-enhanced MRI is the best HCC surveillance test, nor should it replace ultrasonography in cirrhosis patients. The cost-effectiveness of the intervention should be a major consideration in implementing an MRI surveillance program for HCC [10]. Second, our study cannot provide information about the appropriate time interval for MRI surveillance. In our study, MRI was performed not a predetermined protocol, but only considered when USG showed suboptimal quality. Therefore, most of the patients underwent MRI once on average, and at most twice. Therefore, with our current results alone, it is insufficient to determine the MRI interval as a surveillance test. Studies on the MRI time interval as a surveillance test should be additionally conducted in the future. Another limitation of our study is that magnetic resonance machines with different field strengths and coils with different channels might have different imaging effects. In the case of 1.5-T and 3.0-T MRI, it is generally thought that the image quality of 3.0-T MRI better. Similarly, in the case of body coils, if the number of channels increases, the quality of images may improve. To sum up, we think that the image quality can be improved in use of 3.0-T MRI with multiple channels. However, based on the existing literature, we judged that there is no significant difference between 1.5-T and 3.0-T MRI in the diagnostic ability of HCC [43,44]. The HCC detection capability of the well-set MR test using 1.5-T does not appear to be significantly different from the current 3.0-T system. The reason for this is probably because motion related artifacts or susceptibility artifacts tend to increase more at 3.0T. Therefore, we did not conduct a sub-analysis of MRI field strength or different channels in this study.

## 5. Conclusions

In conclusion, intermittent replacement of ultrasonography with Gd-EOB-DTPA-enhanced MRI during ultrasonography-based HCC surveillance could increase the diagnosis of early-stage HCC, a decrease in HCC recurrence risk and an improvement on overall survival compared to the standard surveillance program based on ultrasonography. Yet these findings require further larger-scale prospective studies confirmation.

## Figures and Tables

**Table 1:** Baseline characteristics.

Characteristics	Total (*n* = 421)	USG Group (*n* = 295)	USG+MRI Group (*n* = 126)	*P*
Age >60 years	265 (62.9%)	194 (65.8%)	71 (56.3%)	0.085
Male, *n* (%)	306 (72.7%)	211 (71.5%)	95 (75.4%)	0.486
Etiology				0.257
HBsAg-positive	308 (73.2%)	209 (70.8%)	99 (78.6%)	
Anti-HCV positive	82 (19.5%)	62 (21.0%)	20 (15.9%)	
Others	31 (7.4%)	24 (8.1%)	7 (5.6%)	
Baseline laboratory values				
Total bilirubin, mg/dL	1.4 ± 1.4	1.5 ± 1.5	1.4 ± 1.4	0.438
Albumin, g/dL	3.8 ± 0.6	3.7 ± 0.7	3.8 ± 0.5	0.071
Prothrombin time, INR	1.4 ± 3.1	1.2 ± 0.7	1.9 ± 5.5	0.205
ALT, IU/L	51.6 ± 56.5	50.0 ± 58.9	55.4 ± 50.7	0.362
Creatinine, mg/dL	1.0 ± 0.9	1.0 ± 1.0	0.9 ± 0.2	0.063
Alpha-fetoprotein, ng/mL	10.7 (4.8, 49.6)	10.7 (4.8, 62.3)	10.8 (4.7, 35.9)	0.517
Hemoglobin, g/dL	13.6 ± 5.4	13.5 ± 6.3	13.7 ± 1.8	0.547
Platelets, ×1000/mm^3^	98.0 (68.0, 138.0)	99.0 (70.0, 143.0)	93.0 (63.5, 132.8)	0.133
MELD score	10.2 ± 4.6	10.3 ± 4.0	10.0 ± 5.9	0.665
CTP classification				0.433
A	334 (79.3%)	230 (78.0%)	104 (82.5%)	
B	82 (19.5%)	62 (21.0%)	20 (15.9%)	
C	5 (1.2%)	3 (1.0%)	2 (1.6%)	
BCLC stage *				0.009
0	153 (36.3%)	99 (33.6%)	54 (42.9%)	
A	214 (50.8%)	148 (50.2%)	66 (52.4%)	
B	30 (7.1%)	26 (8.8%)	4 (3.2%)	
C	24 (5.7%)	22 (7.5%)	2 (1.6%)	
Achieving CR after 1st treatment				0.019
Non-CR	274 (65.1%)	203 (68.8%)	71 (56.3%)	
CR	147 (34.9%)	92 (31.2%)	55 (43.7%)	
Surveillance Imaging modality				
Number of USG	8.0 (3.0, 14.0)	9.0 (4.0, 15.0)	6.0 (3.0, 13.0)	0.015
Number of CT	2.0 (1.0, 4.0)	2.0 (1.0, 3.0)	3.0 (2.0, 6.0)	<0.001
Number of MRI	0.5 (0.0, 1.0)	0.0 (0.0, 0.0)	1.0 (1.0, 2.0)	<0.001
Hepatocellular carcinoma risk index	2.7 ± 1.1	2.8 ± 1.1	2.6 ± 1.1	0.217
Follow-up duration (months)	85 (44, 136)	92 (43, 135)	78 (44, 144)	0.727

The data are reported as n (%) for categorical variables or mean ± standard deviation (SD) or median (interquartile range) for continuous variables; * Barcelona Clinic Liver Cancer staging system. Abbreviations: CR, complete response; USG, ultrasonography; MRI, magnetic resonance imaging; HBsAg, hepatitis B surface antigen; HCV, hepatitis C virus; INR, international normalized ratio; ALT, alanine transaminase; MELD, model for end-stage liver disease; CTP, Child-Turcotte-Pugh; V0 = no vascular invasion; N0 = no lymph-node invasion; M0 = no metastases; TNM, tumor-node-metastasis.

**Table 2:** Factors affecting the overall survival.

Factors	Univariate Analysis	Multivariate Analysis	HR (95% CI)
Age (≥60 years)	0.004	0.008	1.87 (1.18, 2.98)
Male	0.117		
Alpha-fetoprotein (≥400 ng/mL)	<0.001	0.076	1.0 (1.0, 1.0)
CTP classification			
A			1
B	<0.001	<0.001	2.85 (1.85, 4.39)
C	<0.001	<0.001	10.27 (2.96, 35.62)
BCLC stage			
0			1
A	0.003	0.022	1.82 (1.09, 3.05)
B	0.004	0.008	2.85 (1.30, 6.23)
C	<0.001	<0.001	12.07 (6.11, 23.85)
Achieving CR after 1st treatment			
Non-CR			1
CR	<0.001	0.022	0.53 (0.31, 0.91)
Surveillance Imaging modality			
USG group			1
USG+MRI group	0.003	0.047	0.56 (0.32, 0.99)

Abbreviations: CTP, Child-Turcotte-Pugh; BCLC, the Barcelona Clinic Liver Cancer staging system; CR, complete response; USG, ultrasound; MRI, magnetic resonance imaging.

**Table 3:** Factors affecting the time to progression.

Factors	Univariate Analysis	Multivariate Analysis	HR (95% CI)
Age (≥60 years)	0.029	0.069	1.30 (0.98, 1.74)
Male	0.465		
Alpha-fetoprotein (≥400 ng/mL)	0.640		
CTP classification			
A			
B	0.083		
C	0.824		
BCLC stage			
0			1
A	0.001	0.003	1.58 (1.16, 2.16)
B	<0.001	<0.001	2.72 (1.61, 4.61)
C	<0.001	<0.001	2.83 (1.59, 5.03)
Achieving CR after 1st treatment			
Non-CR			
CR	0.990		
Surveillance Imaging modality			
USG group			1
USG+MRI group	0.009	0.091	0.76 (0.55, 1.04)

Abbreviations: CTP, Child-Turcotte-Pugh; BCLC, the Barcelona Clinic Liver Cancer staging system; CR, complete response; USG, ultrasound; MRI, magnetic resonance imaging.

## Data Availability

The data that support the findings of this study are available on request from the corresponding author.

## Supplementary Materials

The following supporting information can be downloaded at: https://www.mdpi.com/article/10.3390/biomedicines11020382/s1, Figure S1: Corrected overall survival of patients who were surveilled with USG alone or with USG and Gd-EOB-DTPA-enhanced MRI (unmatched cohort); Figure S2: Standardized mean difference of confounding variables, before and after IPTW; Figure S3: Overall survival and time to progression of patients who were surveilled with USG alone or with USG and Gd-EOB-DTPA-enhanced MRI (propensity score matched cohort); Table S1: Comparison of tumor stage and initial treatment option between two groups; Table S2: Factors identified on Univariate and Multivariate analyses that affect time to progression in HCC patients (IPTW); Table S3: Factors identified on Univariate and Multivariate analyses that affect overall survival in HCC patients (IPTW); Table S4: Demographic and clinical characteristics of patients (propensity score matched cohort); Table S5: Interaction between image modality and tumor stage.
