# Supplementary material for: Adding MRI as a Surveillance Test for Hepatocellular Carcinoma in Patients with Liver Cirrhosis Can Improve Prognosis
Source: Biomedicines. 2023 Jan 27;11(2):382. doi: 10.3390/biomedicines11020382 (PMC9953123; doi:10.3390/biomedicines11020382)
Supplement: Supplementary file 1 [file biomedicines-11-00382-s001.zip › biomedicines-2117043-supplementary.pdf]

Supporting information

Intermittent replacement of ultrasonography by MRI during surveillance improves clinical outcome by sensitive detection of early stage hepatocellular carcinoma

Su Jong Yu, Jeong-Ju Yoo, Dong Ho Lee, Su Jin Kim, Eun Ju Cho, Jeong-Hoon Lee, Se Hyung Kim, Yoon Jun Kim, Jae Young Lee, Jeong Min Lee, Jung-Hwan Yoon

Table of Contents

1. **Supporting Materials and Methods…………………………..……….2**
2. **Supporting Tables…………………………..………………..………….7**
3. **Supporting Figure Legends…………………………………………………16**

Supporting Materials and Methods

Acquisition of USG, CT and MRI Images

USG examination was performed with HDI 3000 and Ultramark 9 systems (Philips Medical Systems, Bothell, WA), an SSD-5500 system (Aloka Co, Ltd, Wallingford, CT), and an Acuson Sequoia 512 system (Siemens Medical Solutions, Mountain View, CA) equipped with 3- to 5-MHz convex transducers.(1)

Quadruple-phase MDCT consisted of pre-contrast, late arterial, portal venous, and equilibrium phases. Liver CT scans were obtained using one of the following commercially available MDCT scanners: a Lightspeed Ultra 8 scanner (GE Healthcare), a Somatom Sensation 16 scanner (Siemens Medical Solutions), or a Brilliance 64 scanner (Philips Medical Systems). CT scans were performed along the craniocaudal direction. The respective scanning parameters used for the 8-, 16-, and 64-MDCT scanners were detector configuration: 8 x 2.5, 16 x 1.5, and 64 x 0.625 mm and slice thickness: 2.5, 3, and 3 mm. The mean acquisition times for each scanner were 4.6, 5.2, and 4.0 seconds. After unenhanced CT was performed, each patient received 90 to 150 mL (1.5 mL/kg of body weight) of nonionic contrast material [Ultravist 370 (iopromide); Schering] through an 18-guage intravenous catheter inserted into a forearm vein using a MK-IV dedicated CT injector (Envision CT; Medrad, Pittsburgh, PA) at a rate of 3 to 5 mL/s for 30 seconds. The scanning delay for the hepatic arterial phase was 15 to 19 seconds (15 seconds for the 8 MDCT scanners, 17 seconds for the 16 MDCT scanner, and 19 seconds for the 64 MDCT scanner) after achieving enhancement of the descending aorta up to 100 HU as measured using a bolus-tracking technique.(2) A 30 to 33 seconds scan delay (30 seconds for the 8 MDCT scanners and 33 seconds for 16–64 CT scanners) following the arterial phase imaging, was used for the portal venous phase imaging. The equilibrium phase images were obtained 3 minutes after starting contrast administration.(3)

Inverse Probability Treatment Weighting (IPTW)

To reduce the effect of selection bias and potential confounding between the two groups, we used inverse probability treatment weighting (IPTW) using propensity score (PS) on the basis of demographic, laboratory, and imaging characteristics of patients (Braitman LE and Rosenbaum PR. Ann Intern Med 2002;137:693-695). With IPTW, each individual is weighted by the inverse probability of receiving the treatment (MR imaging) that they actually received (Curtis LH, et al. Med Care 2007;45:S103-107). To derive PS, the same 14 adjustment variables were used as for the multivariable Cox PH model. There were no missing values for baseline data. In particular, treated individuals (USG+MRI group) are given an IPTW = 1 / PS and the comparison individuals (USG group) are given an IPTW = 1 / (1-PS), where PS is the probability of receiving the treatment. In this way, each group is weighted up to represent the full sample (Curtis LH, et al. Med Care 2007;45:S103-107). This approach, which was implemented to create balance, involved weighting each patient by the inverse of the probability that he or she would be selected for MR imaging or not. To reduce the variability of the IPTW weights and give individuals with extreme weights less influence, we stabilized the weights by multiplying the treatment and comparison weights (separately) by a constant, equal to the expected value of being in the treatment (USG+MRI) or comparison (USG) groups, respectively. It is known that the stabilization reduces the variability of the IPTW weights and reduces the variance of the treatment effect estimates (Harder VS, et al. Psychol Methods 2010;15:234-249). To measure the balancing, we calculated the standardized bias for each measured covariate for the weighted samples. To conduct IPTW analysis, we mainly used "twang" package in R software (version 3.0; http://cran.r-project.org/). Using generalized boosted regression, it automatically computes PS scores and conducts balance checking. Generalized boosted regression includes non-linear effects and interactions in covariates so we can obtain statistically and numerically stable propensity scores (http://CRAN.R-project.org/package=twang).

Propensity Score Matching

We performed propensity score matching for the entire cohort. To fulfill the ignorable intervention-assignment assumption for the propensity score method, we included the following variables for the entire cohort: age; gender; cause of liver disease; serum levels of alanine aminotransferase (ALT), albumin, total bilirubin, and alpha-fetoprotein (AFP); INR; platelet count; ascites; Child-Turcotte-Pugh score; size of the primary HCC nodule on CT images and the presence of a secondary indeterminate lesion on CT images; and Barcelona Clinic Liver Cancer (BCLC) stage as determined by CT. The included variables are expected to influence both the outcome and the intervention. The propensity score was estimated nonparametrically using the "MatchIt" function in the R package "MatchIt". Non-parametric propensity score estimation is useful because we do not have to fit an entirely corrected parametric model between the treatment variables and pretreatment covariates (Ho DE, et al. 2007; 15:199-236). It does not assume any relationship between the treatment and the pretreatment covariates. Matching was performed using the nearest neighbor-matching method, using a caliper width of 0.1 multiplied by the standard deviation for linearly transformed propensity scores (logit-transformation). A nearest neighbor matching of 1:1 selects for each USG+MRI group individual, and identifies the USG group individual showing the smallest distance from the USG+MRI group subject on the logit-transformation scale. Considering the number of subjects in the USG group (n = 377) and USG+MRI group (n = 323), we used 1:1 matching. Figure 4A shows the distribution of the propensity scores for the entire cohort. To evaluate the quality of the matching, we calculated the standardized differences in the mean for group comparisons before and after matching. The following formula was used to calculate continuous or binary pretreatment covariates: $(\mu M - \mu C)/\sigma M$ or $(PM - PC)/\sqrt{PM(1 - PM)}$, where μM, σM and PM are the mean, standard deviation, and proportion for the USG+MRI group, and μC and PC are the mean and proportion for the USG group. If a categorical variable had several levels, we created several dummy variables. In general, it is considered that pretreatment variable balancing is achieved as long as the absolute standardized difference is < 0.20. Figure 4B shows the standardized differences in baseline covariates. No standardized difference for any baseline covariate in

our matches exceeded 0.1. We completed the statistical inference using Cox regression models with robust standard errors and a sandwich covariance matrix estimation, which accounted for the clustering of matched pairs.

Supplementary Table S1. Comparison of tumor stage and initial treatment option between two groups

Characteristics	Total (*n*=421)	USG group (*n*=295)	USG+MRI group (*n*=126)	*P*
Cancer status				0.009
Solitary ≤ 2 cm, V0, N0, M0	224 (53.2%)	143 (48.5%)	81 (64.3%)	
Solitary 2.1 – 3 cm, V0, N0, M0	64 (15.2%)	48 (16.3%)	16 (12.7%)	
Solitary 3.1 – 5 cm, V0, N0, M0	21 (5.0%)	19 (6.4%)	2 (1.6%)	
2–3 nodules, ≤ 3 cm, V0, N0, M0	58 (13.8%)	40 (13.6%)	18 (14.3%)	
Beyond Milan criteria	54 (12.8%)	45 (15.3%)	9 (7.1%)	
AJCC TNM stage[*]				0.205
I	294 (69.8%)	200 (67.8%)	94 (74.6%)	
II	98 (23.3%)	68 (23.1%)	30 (23.8%)	
IIIA	3 (0.7%)	3 (1.0%)	0 (0.0%)	
IIIB	16 (3.8%)	14 (4.7%)	2 (1.6%)	
IIIC	2 (0.5%)	2 (0.7%)	0 (0.0%)	
IVA	4 (1.0%)	4 (1.4%)	0 (0.0%)	
IVB	4 (1.0%)	4 (1.4%)	0 (0.0%)	
BCLC stage[**]				0.009
0	153 (36.3%)	99 (33.6%)	54 (42.9%)	
A	214 (50.8%)	148 (50.2%)	66 (52.4%)	
B	30 (7.1%)	26 (8.8%)	4 (3.2%)	
C	24 (5.7%)	22 (7.5%)	2 (1.6%)	

Treatment modality				0.273
Curative treatments†	220 (52.3%)	148 (50.2%)	72 (57.1%)	
Non-curative treatment‡	188 (44.7%)	136 (46.1%)	52 (41.3%)	
Conservative treatments	13 (3.1%)	11 (3.7%)	2 (1.6%)	

† Curative treatments included liver transplantation, surgical resection, and radiofrequency ablation.

‡ Noncurative treatments included percutaneous ethanol injection, transarterial chemoembolization, systemic chemotherapy, and radiotherapy.

Supplementary Table S2. Factors identified on Univariate and Multivariate analyses that affect time to progression in HCC patients (IPTW)

Factors	Univariate analysis	Multivariate analysis	HR (95% CI)
Age (≥60 years)	0.135		
Male	0.351		
Alpha-fetoprotein (≥400 ng/mL)	0.250		
CTP classification			
A			1
B	0.041	0.161	0.72 (0.46, 1.13)
C	0.668	0.564	0.52 (0.05, 4.80)
BCLC stage			
0			1
A	0.003	0.384	1.16 (0.83, 1.62)
B	<0.001	0.112	1.62 (0.89, 2.95)
C	0.012	0.970	1.01 (0.41, 2.47)
Achieving CR after 1st treatment			
Non-CR			1
CR	<0.001	<0.001	0.00 (0.00-0.00)
Surveillance Imaging modality			
USG group			1
USG+MRI group	0.007	0.024	0.70 (0.51, 0.95)

Abbreviations: CTP, Child-Turcotte-Pugh; BCLC, the Barcelona Clinic Liver Cancer staging system; CR, complete response; USG, ultrasound; MRI, magnetic resonance imaging

Supplementary Table S3. Factors identified on Univariate and Multivariate analyses that affect overall survival in HCC patients (IPTW)

Factors	Univariate analysis	Multivariate analysis	HR (95% CI)
Age (≥60 years)	0.010	0.020	1.77 (1.09, 2.88)
Male	0.237		
Alpha-fetoprotein (≥400 ng/mL)	<0.001	<0.001	1.0 (1.0, 1.0)
CTP classification			
A			
B	0.222		
C	0.054		
BCLC stage			
0			1
A	<0.001	0.001	2.52 (1.44, 4.40)
B	0.004	0.009	3.04 (1.32, 6.98)
C	<0.001	<0.001	16.10 (6.92, 37.47)
Achieving CR after 1st treatment			
Non-CR			1
CR	0.001	0.046	0.54 (0.30, 0.99)
Surveillance Imaging modality			
USG group			1
USG+MRI group	0.015	0.209	0.72 (0.43, 1.19)

Abbreviations: CTP, Child-Turcotte-Pugh; BCLC, the Barcelona Clinic Liver Cancer staging system; CR, complete response; USG, ultrasound; MRI, magnetic resonance imaging

Supplementary Table S4. Demographic and clinical characteristics of patients (propensity score matched cohort)

Characteristics	Total (*n*=252)	USG group (*n*=126)	USG+MRI group (*n*=126)	*P*
Age > 60 years	139 (55.2%)	68 (54.0%)	71 (56.3%)	0.800
Male, *n* (%)	189 (75.0%)	94 (74.6%)	95 (75.4%)	1.000
Etiology				0.670
HBsAg-positive	192 (76.2%)	93 (73.8%)	99 (78.6%)	
Anti-HCV positive	44 (17.5%)	24 (19.0%)	20 (15.9%)	
Others	16 (6.3%)	9 (7.1%)	7 (5.6%)	
Baseline laboratories				
Total bilirubin, mg/dL	1.4 ± 1.6	1.4 ± 1.7	1.4 ± 1.4	0.645
Albumin, g/dL	3.8 ± 0.6	3.8 ± 0.8	3.8 ± 0.5	0.472
Prothrombin time, INR	1.5 ± 3.9	1.2 ± 0.2	1.9 ± 5.5	0.168
ALT, IU/L	50.3 ± 45.5	45.1 ± 39.2	55.4 ± 50.7	0.070
Creatinine, mg/dL	1.0 ± 1.0	1.1 ± 1.5	0.9 ± 0.2	0.295
Alpha-fetoprotein, ng/mL	10.9 [4.7, 41.0]	11.1 [4.8, 59.1]	10.8 [4.7, 35.9]	0.705
Hemoglobin, g/dL	13.9 ± 6.7	14.0 ± 9.4	13.7 ± 1.8	0.716
Platelet, x1000/mm^3	102.5 [70.8, 139.0]	109.0 [78.2, 149.5]	93.0 [63.5, 132.8]	0.011
MELD score	10.0 ± 5.0	10.0 ± 3.8	10.0 ± 5.9	0.927
CTP classification				0.828
A	210 (83.3%)	106 (84.1%)	104 (82.5%)	
B	39 (15.5%)	19 (15.1%)	20 (15.9%)	

C	3 (1.2%)	1 (0.8%)	2 (1.6%)	
Cancer status				<0.001
Solitary ≤ 2 cm, V0, N0, M0	134 (53.2%)	53 (42.1%)	81 (64.3%)	
Solitary 2.1 – 3 cm, V0, N0, M0	42 (16.7%)	26 (20.6%)	16 (12.7%)	
Solitary 3.1 – 5 cm, V0, N0, M0	14 (5.6%)	12 (9.5%)	2 (1.6%)	
2–3 nodules, ≤ 3 cm, V0, N0, M0	32 (12.7%)	14 (11.1%)	18 (14.3%)	
Beyond Milan criteria	30 (11.9%)	21 (16.7%)	9 (7.1%)	
AJCC TNM stage*				-
I	185 (73.4%)	91 (72.2%)	94 (74.6%)	
II	54 (21.4%)	24 (19.0%)	30 (23.8%)	
IIIA	1 (0.4%)	1 (0.8%)	0 (0.0%)	
IIIB	9 (3.6%)	7 (5.6%)	2 (1.6%)	
IIIC	1 (0.4%)	1 (0.8%)	0 (0.0%)	
IVA	2 (0.8%)	2 (1.6%)	0 (0.0%)	
IVB	0 (0.0%)	0 (0.0%)	0 (0.0%)	
BCLC stage**				0.063
0	97 (38.5%)	43 (34.1%)	54 (42.9%)	
A	131 (52.0%)	65 (51.6%)	66 (52.4%)	
B	15 (6.0%)	11 (8.7%)	4 (3.2%)	
C	9 (3.6%)	7 (5.6%)	2 (1.6%)	
Achieving CR after 1st treatment				0.069
Non-CR	157 (62.3%)	86 (68.3%)	71 (56.3%)	

CR	95 (37.7%)	40 (31.7%)	55 (43.7%)	
Image modality				
Number of USG	8.0 [3.0, 14.0]	9.0 [4.0, 15.0]	6.0 [3.0, 13.0]	0.015
Number of CT	2.0 [1.0, 4.0]	2.0 [1.0, 4.0]	3.0 [2.0, 6.0]	<0.001
Number of MRI	0.0 [0.0, 1.0]	0.0 [0.0, 0.0]	1.0 [1.0, 1.0]	<0.001
Hepatocellular carcinoma risk index	2.7 ± 1.1	2.8 ± 1.1	2.6 ± 1.1	0.217
Follow up duration (months)	85.0 [44.0, 136.0]	92.0 [43.0, 135.0]	78.5 [44.0, 144.0]	0.727

Supplementary Table S5. Interaction between image modality and tumor stage

Factors	Univariable		Mulivariable	
	HR (95% CI)	P-value	HR (95% CI)	P-value
Age ≥ 60 years	1.88 (1.16, 3.07)	0.010	1.73 (1.07, 2.78)	0.024
Male, *n (%)*	0.76 (0.49, 1.18)	0.237		
Alpha-fetoprotein ≥ 400ng/mL	1.0 (1.0, 1.0)	<0.001	1.0 (1.0, 1.0)	<0.001
CTP classification				
A	1			
B	1.37 (0.82, 0.96)	0.222		
C	5.87 (0.82, 0.96)	0.054		
Achieving CR after 1st treatment				
Non-CR	1		1	
CR	0.36 (0.20, 0.64)	0.001	0.54 (0.30, 0.97)	0.040
Surveillance Imaging modality				
USG group	1		1	
USG+MRI group	0.25 (0.06, 1.09)	0.066	0.25 (0.15, 0.42)	<0.001
BCLC stage				
0	1		1	
A	1.70 (0.96, 3.00)	0.065	1.55 (1.00, 2.38)	0.046
B	2.70 (1.15, 6.35)	0.022	2.49 (1.22, 5.08)	0.012
C	14.37 (6.18, 33.42)	<0.001	10.83 (5.56, 21.08)	<0.001
BCLC stage * imaging modality				
USG+MRI group * stage A	3.72 (0.79, 17.44)	0.095	3.91 (2.25, 6.79)	<0.001
USG+MRI group * stage B	0.0 (0.0, 0.0)	<0.001	0.0 (0.0, 0.0)	<0.001
USG+MRI group * stage C	3.39 (0.48, 23.89)	0.220	3.76 (1.53, 9.23)	0.004

Supplementary Figure S1. Corrected overall survival of patients who were surveilled with USG alone or with USG and Gd-EOB-DTPA-enhanced MRI (unmatched cohort)

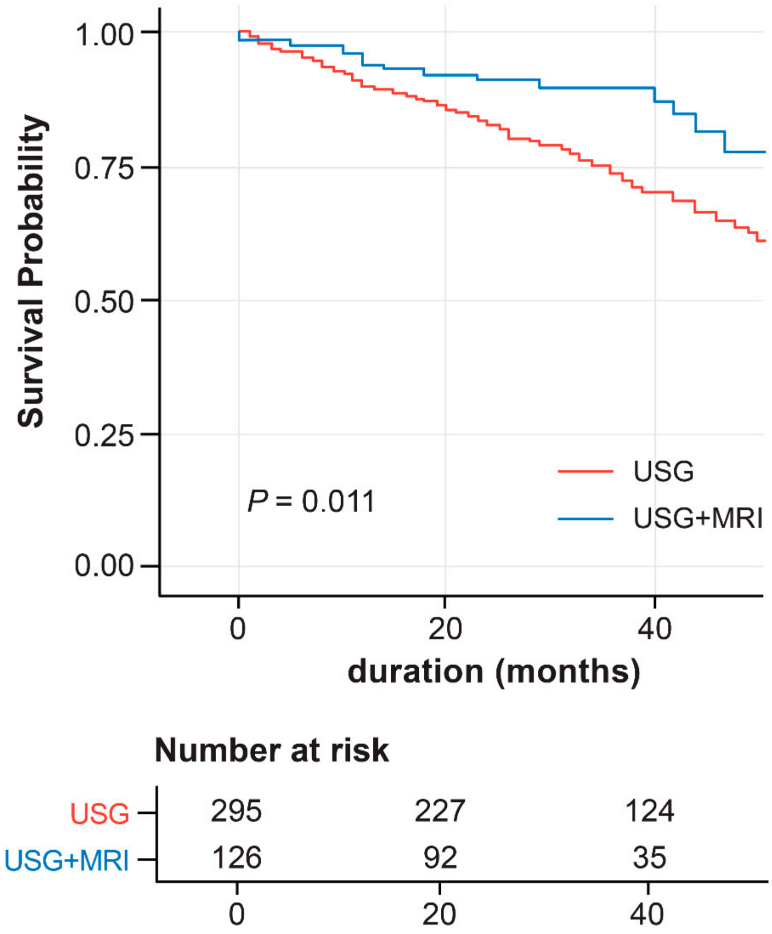

Supplementary Figure S2. Standardized mean difference of confounding variables, before and after IPTW

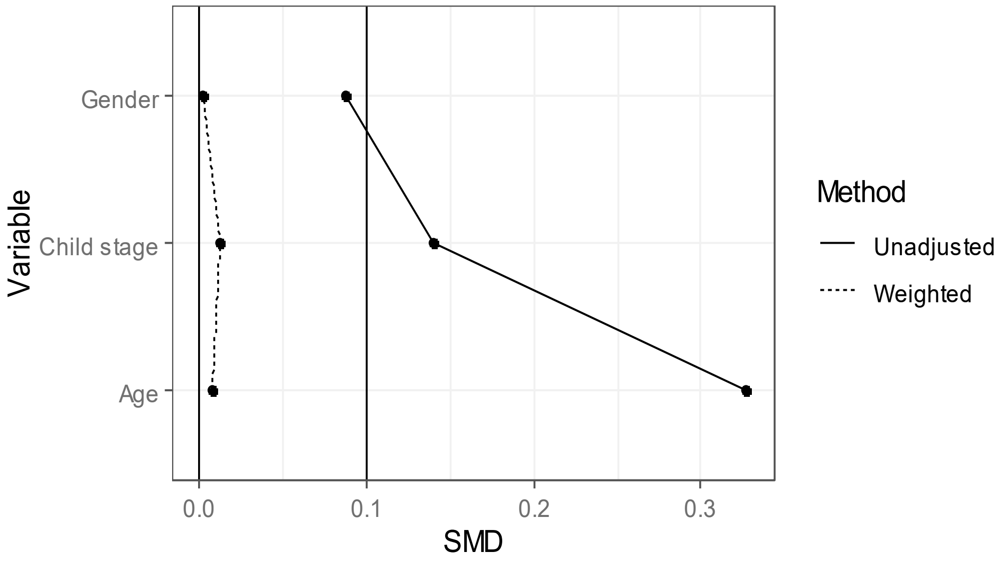

Supplementary Figure S3. Overall survival and time to progression of patients who were surveilled with USG alone or with USG and Gd-EOB-DTPA-enhanced MRI (propensity score matched cohort)

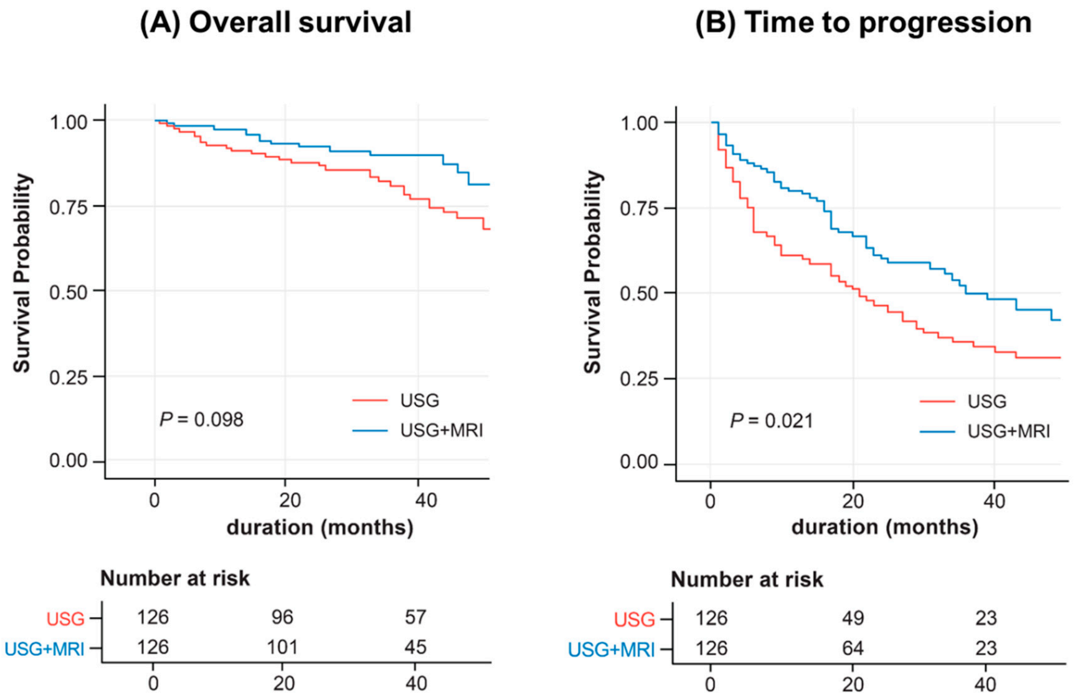

References

1. Kim NR, Kim SH, Lee JM, Lee KH, Kim YJ, An SK, Jung AY, et al. Sonographic features of an intraductal polypoid mass: differentiation between hepatocellular carcinoma and intraductal cholangiocarcinoma. J Ultrasound Med 2004;23:1283-1291.

2. Itoh S, Ikeda M, Achiwa M, Satake H, Iwano S, Ishigaki T. Late-arterial and portal-venous phase imaging of the liver with a multislice CT scanner in patients without circulatory disturbances: automatic bolus tracking or empirical scan delay? Eur Radiol 2004;14:1665-1673.

3. Sun HY, Lee JM, Shin CI, Lee DH, Moon SK, Kim KW, Han JK, et al. Gadoxetic acid-enhanced magnetic resonance imaging for differentiating small hepatocellular carcinomas (< or =2 cm in diameter) from arterial enhancing pseudolesions: special emphasis on hepatobiliary phase imaging. Invest Radiol 2010;45:96-103.
